# Motor cortical activity changes during neuroprosthetic-controlled object interaction

**DOI:** 10.1038/s41598-017-17222-3

**Published:** 2017-12-05

**Authors:** John E. Downey, Lucas Brane, Robert A. Gaunt, Elizabeth C. Tyler-Kabara, Michael L. Boninger, Jennifer L. Collinger

**Affiliations:** 1 Department of Bioengineering, University of Pittsburgh, Pittsburgh, Pennsylvania USA; 2 Center for the Neural Basis of Cognition, Pittsburgh, Pennsylvania USA; 3 School of Medicine, University of Pittsburgh, Pittsburgh, Pennsylvania USA; 4 Department of Neurological Surgery, University of Pittsburgh, Pittsburgh, Pennsylvania USA; 5 Department of Physical Medicine and Rehabilitation, University of Pittsburgh, Pittsburgh, Pennsylvania USA; 6 VA Pittsburgh Healthcare System, Pittsburgh, Pennsylvania USA

## Abstract

Brain-computer interface (BCI) controlled prosthetic arms are being developed to restore function to people with upper-limb paralysis. This work provides an opportunity to analyze human cortical activity during complex tasks. Previously we observed that BCI control became more difficult during interactions with objects, although we did not quantify the neural origins of this phenomena. Here, we investigated how motor cortical activity changed in the presence of an object independently of the kinematics that were being generated using intracortical recordings from two people with tetraplegia. After identifying a population-wide increase in neural firing rates that corresponded with the hand being near an object, we developed an online scaling feature in the BCI system that operated without knowledge of the task. Online scaling increased the ability of two subjects to control the robotic arm when reaching to grasp and transport objects. This work suggests that neural representations of the environment, in this case the presence of an object, are strongly and consistently represented in motor cortex but can be accounted for to improve BCI performance.

## Introduction

The capabilities of the human arm and hand allow us to interact with our environment in a multitude of ways that are crucial for activities of daily living. Our ability to reach out for a cup of coffee, grasp and turn a door knob, swing a hammer, or button a shirt, are all accomplished effortlessly with the same apparatus. The loss of arm and hand function therefore leads to a severe loss of independence. This sense of loss is reflected in studies of people with spinal cord injury who report that restoration of arm and hand function is a top priority^1–3^. For injuries like spinal cord injuries and amputation, assistive technology is required to replace the functions previously performed by the arm and hand. To address this issue, brain-computer interfaces (BCI) that enable control of robotic arms are being developed^4–8^.

A great deal is known about how primary motor cortex (M1) encodes movement parameters during free reaching^9–18^, and BCI control of robotic devices in both animals^19^ and humans^4–8,20^ is based on this understanding. We have previously shown that while using a BCI, a person can continuously and simultaneously control 10 degrees of freedom of an anthropomorphic robotic arm including positioning and orienting the hand and wrist, as well as multiple grasp postures^8^. Although the BCI enabled consistent and skilled performance of movements performed in open space, we noted difficulties when the subject tried to grasp objects^8^. This often manifested as an unwanted movement away from the target object or the inability to execute a grasp around an object (Supplementary Video 1). If the object was removed from the workspace, the ability to accurately control the arm was restored.

Because BCI arm movement results from a defined transformation of the recorded neural firing rates, a reduction in performance implies that the M1 activity was different when an object was present in the workspace. This led us to modify our calibration paradigm to account for this^8^. Initially we had estimated the relationship between neural firing rates and kinematics while the subject attempted to move the robotic arm in open space^8^. To mitigate the challenges with object grasping, we began to calibrate in a virtual-reality environment where the participant could observe a virtual arm grasping and transporting virtual objects. Incorporating virtual objects into our calibration paradigm helped to overcome the challenges with grasping simple objects and enabled the participant to complete functional assessments of performance that she had previously been unable to perform consistently^8^. However, there remained a gap in our understanding of how neural activity changes during object manipulation as compared to simple kinematic movements.

In order for a BCI-controlled robotic device to truly replace function, it must enable reliable and safe object manipulation across a variety of environmental conditions. However, the neural control of hand movements and object manipulation has been less studied than reaching^9–18^. Some studies in non-human primates have reported that M1 encodes information about the size and shape of the object that is being grasped^21–23^. However, other work indicates that motor cortex encodes information related to hand shaping rather than information about the object itself^24–26^. To expand on previous studies of free reaching, or reaching to grasp^9–18,21–26^, we sought to study the differences in M1 activity between reaches involving objects and those that do not involve objects.

In this study, we report findings in two human intracortical BCI users as they reach and grasp an object with a robotic arm or generate the same movement without an object in the workspace. We found that a large proportion of M1 neurons increased their firing rate while the hand approached an object, but not while performing the same movement without an object in the workspace. Task performance suffered since this change in neural firing rates was unaccounted for in our BCI decoding algorithm. However, by implementing an online scaling method to account for object-related changes in M1 firing rates, we could restore the subjects’ ability to perform reaching and grasping tasks with the BCI.

## Results

Two subjects with tetraplegia had intracortical microelectrode arrays (Blackrock Microsystems, Inc., Salt Lake City, Utah) implanted in motor cortex as part of a study covered by an Investigational Device Exemption (See Methods for details). This study consisted of two primary experiments: (1) Quantification of M1 activity during BCI-controlled reach and grasp movements with and without an object in the workspace and (2) Development and evaluation of an online scaling paradigm designed to account for changes in neural firing rate to improve the subjects’ ability to interact with objects.

### Object presence impairs BCI control by increasing firing rates

The ‘object interaction task’ was used to determine how the presence of an object modulates neural activity during reaching and grasping. Each subject completed two conditions: (1) Reaching to and grasping a cylindrical object (5 cm diameter, 14 cm height) and (2) Reaching to the same position in space and closing the hand in the absence of a physical object in the workspace. Both participants experienced a decrease in success rate on the object interaction task when an object was present in the workspace (Supplementary Video 2). Subject 1 successfully completed 82% of trials when no object was present (25/30 trials, across 6 days). In contrast, she only successfully completed the task 60% of the time if an object was present in the target region (18/30 trials, across 6 days). Subject 2 successfully completed 79% of trials when no object was present (55/70 trials, across 2 days), but the success rate decreased to 21% (15/70 trials, across 2 days) when an object was present.

**Figure 1:**
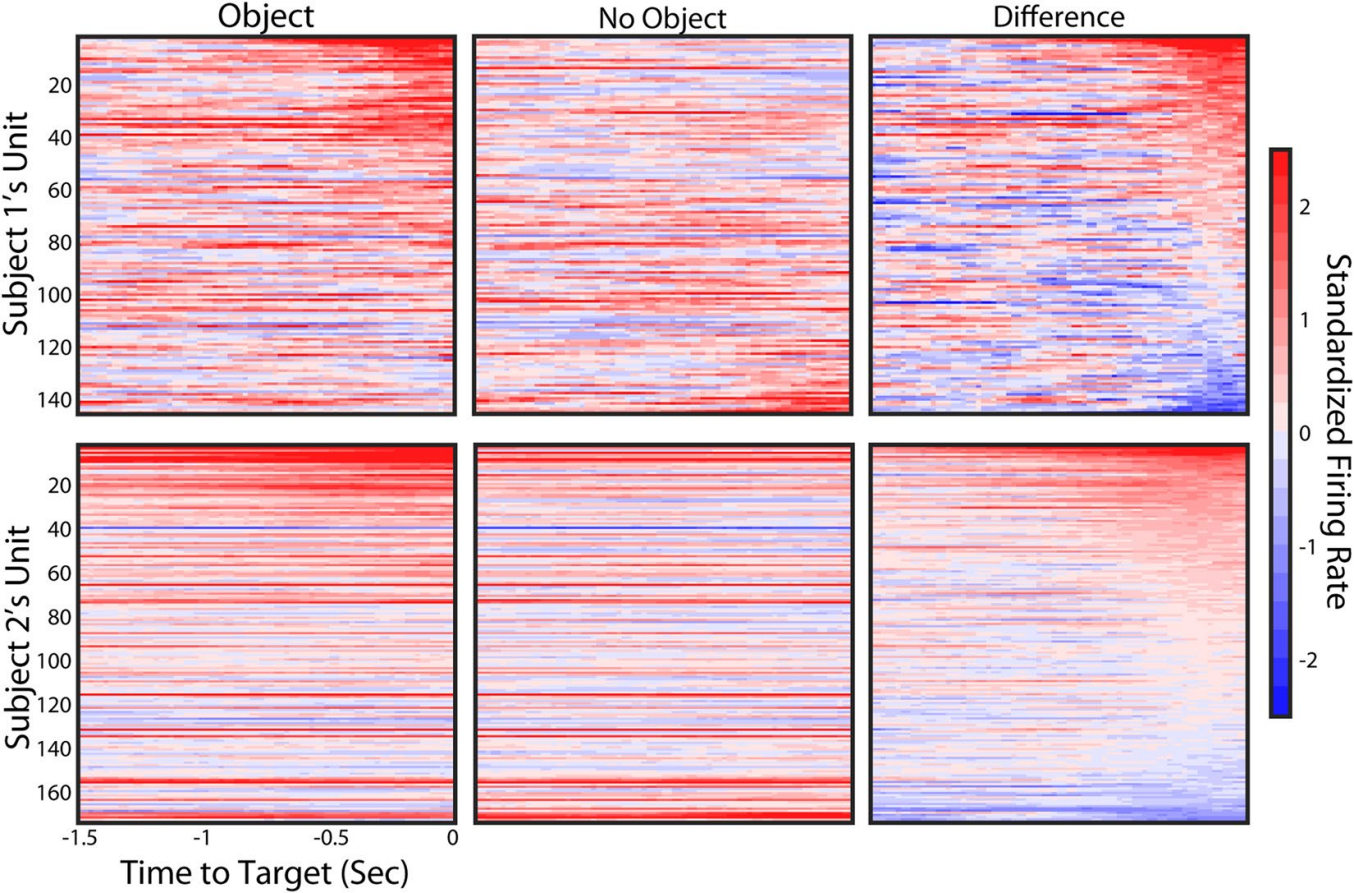
Neural response to object interaction task. Standardized firing rates from Subject 1 (top row) and Subject 2 (bottom row) averaged across trials from a single day of testing are shown for each recording channel. The channels are sorted according to the difference in standardized firing over the last 250 ms of the reach phase between the object and no object conditions with the units showing the highest difference at the top of each plot. On the testing day that is shown, Subject 1 completed 5 trials per condition and Subject 2 completed 50 trials per condition.

The decrease in task performance was accompanied by changes in population firing rate. Figure 1 shows that an increase in firing rate was measured on a majority of channels as the hand moves closer to the object (i.e., the end of the reach phase). Data are presented as a standardized firing rate where measured data is normalized to the mean and standard deviation of the firing rate recorded during calibration (See Methods for details). As would be expected during BCI control, Fig. 1 shows that on a representative day of testing, many of the channels measured an increase or decrease in firing rate as compared to baseline, which is to be expected because each unit has a different preferred direction and all reaches were to the left. The ‘Difference’ plot on the far right in Fig. 1 highlights the object-specific changes in firing rate that affected more than half of the recorded units. These units fired faster as the hand got closer to the object when compared to the firing rates that were measured during the same kinematic movement without an object in the workspace. Because the movement kinematics were the same whether or not an object was present, the difference plot shows no change in firing rate for units that are unaffected by object presence. During the 250 ms before the end of the reach phase 69.8% of Subject 1’s units and 68.4% of Subject 2’s units fired faster when an object was present across all test days (i.e. positive values at the right side of the difference plot in Fig. 1). To give a sense of the strength of this effect, we define units that are highly-sensitive to object presence as those that have a change in firing rate between the ‘object’ and ‘no object’ conditions that is greater than 1 standard deviation above or below the mean firing rate during decoder calibration. Subjects 1 and 2 had 13.7% and 10.5% of their units respectively show a high sensitivity to the object that resulted in an increase in firing rate. Only 3.6% of Subject 1’s units and 1.7% of Subject 2’s units exhibited a high sensitivity to the object resulting in a decrease in firing rate.

**Figure 2:**
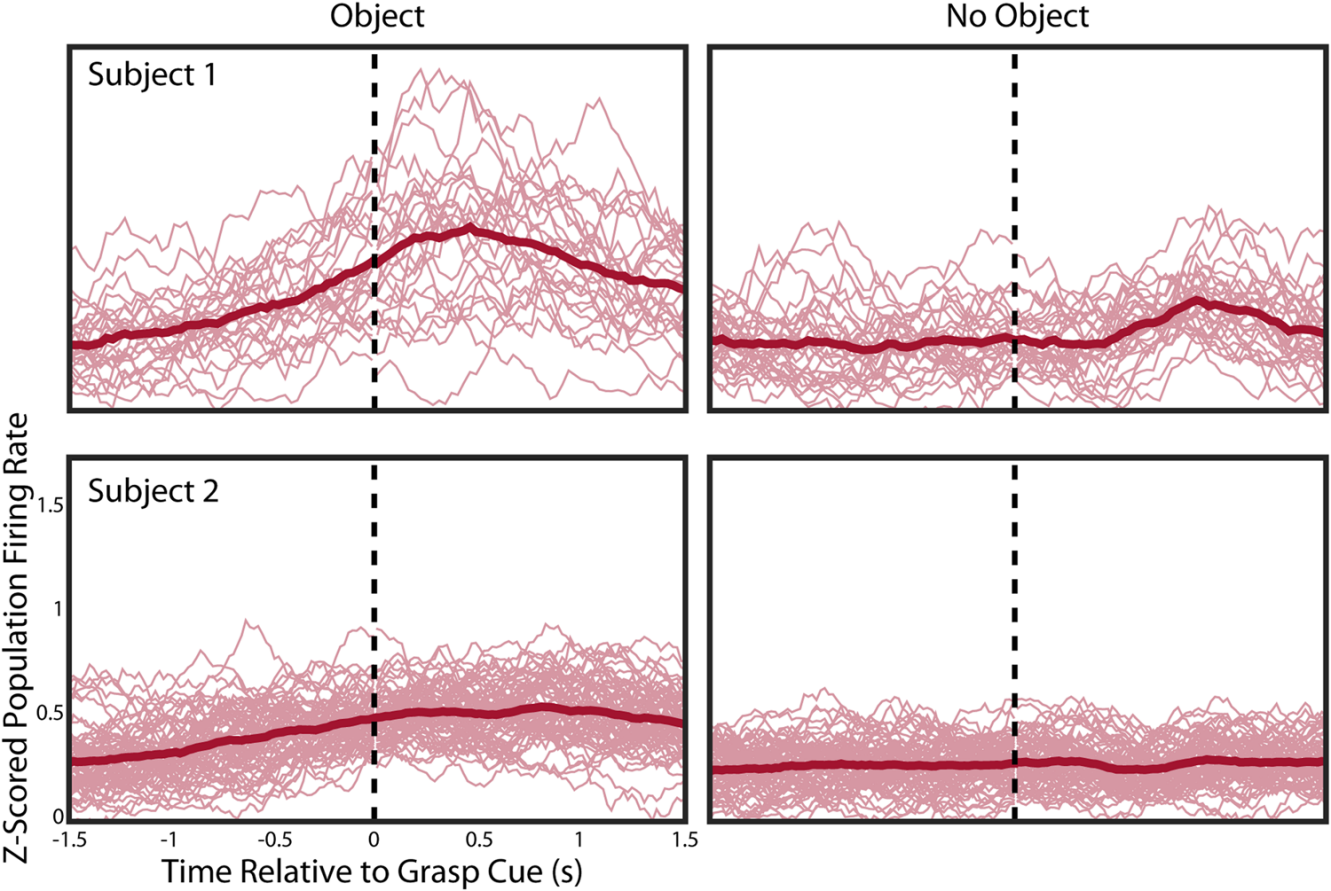
Population firing rate trajectories. The thin faded lines show the z-scored population firing rate for the 1.5 seconds before and after the hand reached the target (vertical dashed line) for every trial across all testing days (6 for Subject 1, and 2 for Subject 2). The bold line shows the average z-scored population firing rate for each condition. Both subjects showed increased population firing before object contact, while neither showed increasing firing rates while reaching towards the empty target region.

The increase in standardized population firing rate was consistently observed in both participants across trials completed over multiple days as shown in Fig. 2. The population firing rate increased throughout the reach as the hand moved closer to the object. When no object was present, the standardized population firing rate remained nearly constant during the reach phase. In both participants, there was a significant difference between the standardized population firing rate during the reach phase, as measured during the last 250 ms of the reach phase, when an object was present compared to when the subject performed the same movement without an object in the workspace (p < 0.001, Wilcoxon Rank Sum). When reaching to an object, Subject 1’s median population firing rate during the last 250 ms of the reach phase across 30 reaches was 0.66 standard deviations higher than the mean firing rate measured during calibration in the VR environment, but only 0.34 standard deviations higher if no object was present. This is a difference equivalent to an increase in firing rate of 0.24 Hz in every unit in the population for Subject 1 when an object is present. Similarly, Subject 2’s median population firing rate across 70 reaches was 0.48 standard deviations higher than baseline when an object was present, and only 0.27 standard deviations higher without an object. This is a difference equivalent to an increase in firing rate of 0.27 Hz in every unit in the population for Subject 2. The population firing rate continues to increase for approximately 0.5 seconds after the cue to grasp. We also observed a slight increase in firing rate at approximately 0.75–1 seconds into the grasp phase, which is approximately when the hand would close although grasp timing relative to the onset cue was variable across trials (Fig. 2).

Additionally, we investigated whether the change in population firing rate was related to the subject’s plan to grasp the object, or simply due to the object being present. To do so, Subject 1 completed 30 trials, across 6 days, of an additional condition where she reached to position the hand at the target, but simply kept the hand in an open position. When the subject did not intend to grasp, there was still a significant difference in population-level firing rate between the object and no object trials during the last 250 ms of the reach phase (0.47 standard deviations above baseline for trials with an object, vs. 0.34 for trials with no object, p = 0.02, Wilcoxon rank-sum test). We also confirmed that when no object was present in the workspace, population firing rate at the end of the reach was the same whether or not the subject intended to grasp or keep the hand open (0.34 standard deviations above baseline, p = 0.97, Wilcoxon rank-sum test).

### Online population-level scaling improves BCI object grasping

The large increase in firing rate that occurred when an object was present provides an explanation for the decrease in performance during reaches to objects as compared to movements performed in open space. This population-level increase in firing rate (shown in Fig. 2) violates the neural encoding model (See Equation 1 in Methods for additional detail) used to create the decoders used in this BCI system. In the encoding model, the firing rate for each channel is a linear function of the desired movement velocity, and it is assumed that the coefficients relating intended velocities to firing rate are independent^8,16^. Here we found that a large proportion of the recorded units had increased firing rates when an object was present. This indicates that the neurons are sensitive to a factor other than velocity, and that the response to this factor is not independent across the population.

To adjust for this population-level change in firing rates, we implemented a scaling factor that operated during BCI control without knowledge of the task being performed. The scaling factor was calculated by computing the average population firing rate over the most recent 300 ms and dividing that by the average population firing rate observed during decoder calibration (Equation 2 in Methods). Each channel’s firing rate was then divided by this scaling factor before being decoded. As with the changes in population firing rate shown in Fig. 2, the scaling factor increased as the hand approached the object. For trials with an object, the median scaling factor increased from 1.47 (1.29–1.62 IQR) 1.5 seconds before the end of the reach to 1.54 (1.43–1.71 IQR) at the end of the reach. For Subject 2 the median scaling factor started at 1.03 (0.98–1.07 IQR) and increased to 1.10 (1.06–1.15 IQR) at the end of the reach phase.

After implementing online scaling, Subject 2’s success rate on the object interaction task increased from 21% to 56% when an object was in the target region (p = 0.0012, Wilcoxon rank-sum, Supplementary Video 3). He was successful on 77% of trials with scaling and 79% of trials without scaling when no object was present, showing that scaling did not impair performance (p = 0.94, Wilcoxon rank-sum). Subject 1 had a success rate of 57% without scaling and 63% with scaling when a cylinder was in the target region. While not significantly different (p = 0.77, Wilcoxon rank-sum), this may be because her performance with objects was better than Subject 2 to begin with. She had been practicing functional tasks involving objects for months without scaling prior to this experiment and may have learned to modulate her neural activity to overcome any object-related changes in firing rate. She was successful on 93% of trials with scaling and 63% of trials without scaling when no object was present (p = 0.18, Wilcoxon rank-sum).

**Figure 3:**
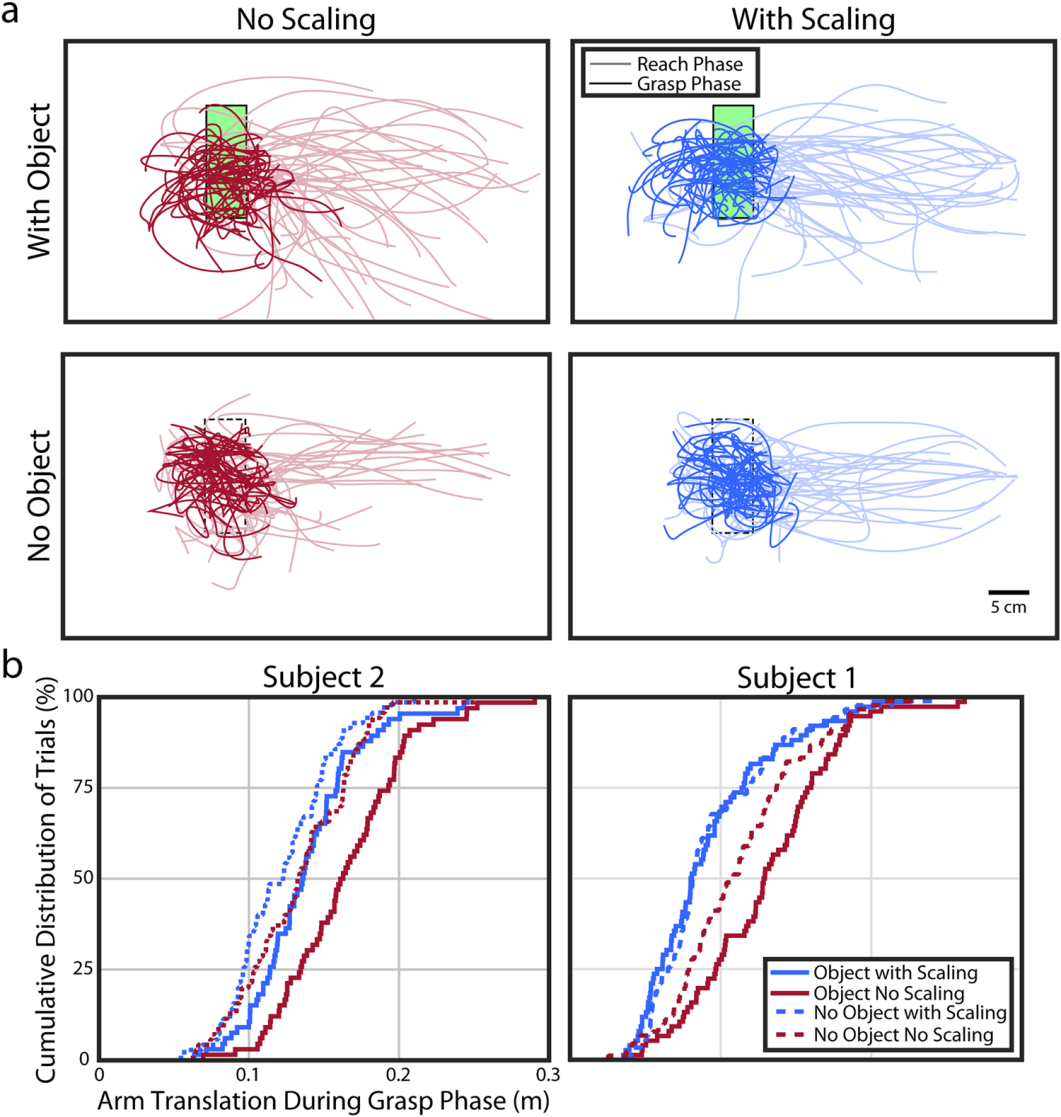
Movement trajectories near the target across all testing days. (**a**) The endpoint position of the arm is shown for the 1.5 seconds before (faded color) and after (saturated color) Subject 2 guided the hand to the target region. The left column shows reaches without scaling. The right column shows reaches with scaling. With scaling, the reach movement is much more consistent and there is less movement once the hand reaches the target. (**b**) The cumulative distribution of the path length in the 1.5 seconds after the hand reached the target and the subject was cued to grasp. Once the hand reaches the target no more arm movement is necessary, so longer paths (conditions that are further right in the plot) demonstrate less ability to stabilize the hand.

Upon examination of the reach kinematics, we found that subjects had an improved ability to stabilize the hand near the object. We quantified the movement of the arm for the 1.5 seconds after the hand reached the target and the subject was cued to grasp. In a perfect case, no translation of the arm (measured at the endpoint near the palm) would be observed as the subject closed the hand to a grasped position. Both participants showed a significant decrease in the amount of arm translation when scaling was implemented (both p < 0.005, Wilcoxon rank-sum). Figure 3a illustrates the arm endpoint position for reaches to an object and for movements without an object in the workspace for trials performed by Subject 2 with and without scaling. When Subject 2 performed reaches with an object present, the arm had a median trajectory length of 0.16 meters in the 1.5 seconds after the cue to grasp without scaling, but only 0.13 meters with scaling (p = 1.2 × 10^−4^, Wilcoxon rank-sum). Similarly, Subject 1 had a median trajectory length of 0.12 meters without scaling and 0.08 meters with scaling (p = 1.3 × 10^−5^, Wilcoxon rank-sum) when reaching towards an object. When no object was present, Subject 2 had a median trajectory of 0.13 meters without scaling and 0.12 meters with scaling (p = 0.068, Wilcoxon rank-sum). Subject 1 had a median trajectory length of 0.09 meters without scaling and 0.08 meters with scaling (p = 0.015, Wilcoxon rank-sum). Again, these results show an improvement in performance with scaling when an object is present, but no detriment to performance in the absence of an object.

Finally, the subjects performed an object transport task to provide a more functional assessment of the effect of scaling in a task related to activities of daily living. The goal of the task was to pick up a cylindrical object (15.5 cm tall, 4.5 cm diameter, with a weighted base) on one side of a table, transport it across a 20 cm wide zone on the table, and place it on the other side. An experimenter immediately picked up the object and placed it back in the starting position. The subject had to transport the object as many times as possible in 2 minutes. Subject 1 showed a significant improvement in performance when scaling was used, increasing her object transfer rate over three-fold (p = 0.0273, Wilcoxon rank-sum, Table 1, Supplementary Video 4). Subject 2 also showed significant improvement when scaling was used, increasing his object transfer rate by 14% (p = 0.043, Wilcoxon rank-sum, Table 1, Supplementary Video 5).

**Table 1:** Average number object transfers per minute with and without scaling.

	Transfers Per Minute	
	With Scaling	Without Scaling
Subject 1	1.09 (1.09)	0.35 (0.66)
Subject 2	5.28 (1.21)	4.65 (1.27)

The mean number of transfers per minute (and standard deviation) was computed from 17 two-minute object transfer trials across 6 days for Subject 1. Subject 2 completed 20 trials across 3 days.

## Discussion

Grasping is a complex task that engages multiple brain areas^27,28^. Here we observed that neurons in M1 react to the presence of an object. We implemented a simple method to recognize and account for the changes in population firing rate during BCI control without knowledge of the task or environment. This method of online scaling increased BCI performance during robotic arm control during reaches to objects while not impairing movements without an object in the workspace, essentially improving the reliability and generalizability of the BCI.

We first noticed difficulties with grasping objects under BCI-control during the early arm control experiments, but they became particularly problematic during higher degree-of-freedom control experiments^8^. The nature of the problematic movements would differ from day to day, but were typically consistent across trials using the same decoder. When decoders were calibrated without objects in the workspace, our first participant consistently had difficulty interacting with objects. When virtual objects were introduced into the calibration process the subject appeared to overcome this difficulty and completed functional reaching and grasping tasks reliably during eleven testing sessions across more than a month^8^. As experiments continued more than a year after the introduction of virtual objects, we again began to observe unintentional movements while interacting with objects, even with decoders calibrated with virtual objects. Complications with object interaction within the realm of BCI-controlled prosthetics have not been widely reported or well characterized. Only a few studies have attempted tasks involving object grasping^5,6^. However these have typically been limited to simple tasks that are the same as the calibration procedure or tasks with fewer degrees of freedom, which may be less susceptible to population-level changes in firing rate due to the redundancy of the recorded population relative to the task kinematics.

In this study, we explored a solution for dealing with changes in firing patterns seen with a BCI-controlled robotic arm during object manipulation. This approach involves scaling the firing rates before decoding them to generate movement commands. This scaling removed the correlated increase in firing rates across the recorded population, and improved the ability of the subjects to interact with objects. This population-wide approach had the benefit of being immediately generalizable between days, not requiring calibration each day like a unit-specific method would. Others have attempted to address recording nonstationarities^29–32^, which can be defined as unexpected changes that occur in the statistical characteristics of neural signals. However, these studies largely explore issues with reliability in control over minutes to hours, rather than the object-related phenomena observed here, which occurs on a time scale of hundreds of milliseconds. Methods related to recording nonstationarities primarily seek to compensate for changes in the identity of the recorded neurons, because the loss of a recorded neuron can greatly disrupt the decoding model. In contrast, our work seeks to temporarily adjust for an expected change during a common type of activity. Online scaling may also prove useful in other conditions where population-level firing rate is modulated by a factor other than object presence, for instance attentiveness^33^.

Many questions remain around the neurological origin of the apparent object-sensitivity observed in M1. While not tested here, we speculate that the changes in firing rate reflect preparation for object contact, grasp and manipulation, which requires that the hand and arm be prepared to deal with unexpected sensory input or perturbations. As an example, people often exhibit increased muscle co-contraction when preparing to lift an object with unknown characteristics, such as weight^34^. Future studies should manipulate object properties and task requirements (such as level of precision) in order to better understand whether the observed firing rate changes are modulated by the task.

At a systems level, we suspect that inputs arising from other areas in the visuomotor pathway contribute to the changes in population firing rate in M1 during BCI control. Studies of anterior intraparietal cortex (AIP), ventral premotor cortex (PMv), and M1 have shown that explicit object information is represented in AIP during movement planning and PMv during planning and execution^25,26,35–37^. These studies did not observe explicit object information in M1, but that may be because they did not compare activity to recordings during identical movements without an object in the workspace. Other studies that have shown that M1 contains information about objects such as size and shape, however these differences could also be due to the fact that different hand kinematics are used for each object^21–23^. Further research into the visuomotor pathway, and the computations within and between the cortical areas, will improve our understanding of the interaction of objects and motor control.

An emerging understanding of population dynamics in M1 may also help to explain this firing rate behavior that deviates from the expectations of our linear model for firing rates^38,39^. A dynamic network model for decoding^40^ may naturally avoid the problems that a generalized increase in population firing rate causes for decoders that enforce a linear relationship between firing rates and kinematics like the one used in this work.

It is also worth investigating whether this sensitivity to objects is only present during neuroprosthetic arm use, perhaps due to a lack of proprioceptive or tactile feedback, or whether it is present during native arm movements as well. Ongoing studies of BCI-controlled functional electrical stimulation^41–43^ provide a unique opportunity to study in humans whether the object-sensitivity of M1 units is specific to robotic-arm control or whether it is a natural response to the task environment. While future work is needed to investigate the mechanisms leading to the observed changes in M1 firing, we have presented a simple method for enabling more generalizable BCI control that accounts for object-related challenges.

## Methods

This study was conducted under Investigational Device Exemptions from the U.S. Food and Drug Administration for the study of intracortical BCI (NCT01364480 and NCT01894802). The studies were approved by the Institutional Review Boards at the University of Pittsburgh and the Space and Naval Warfare System Center Pacific. All procedures were conducted in accordance with the policies and guidelines associated with these approvals.

### Study participants

Two participants completed this study to investigate how M1 activity was impacted by the presence of an object during reaching and grasping movements. Informed consent was obtained from both participants prior to completion of any study-related procedures. Subject 1 was a 52-year-old (at time of implant) woman with spinocerebellar degeneration resulting in motor complete tetraplegia at the C4 level with some preserved sensation^44^. Two 96-channel intracortical microelectrode arrays were implanted in the hand and arm region of her primary motor (M1) cortex. She participated in 9 sessions, occurring between 795 and 850 days after implantation. Subject 2 was a 28-year-old (at time of implant) male with tetraplegia due to a C5 motor/C6 sensory ASIA B spinal cord injury^45^. Subject 2 had two 88-channel intracortical microelectrode arrays implanted in the hand and arm region of M1. He also had intracortical microelectrode arrays implanted in finger-related areas of somatosensory cortex, though they were not used in this experiment. Results related to intracortical microstimulation through the electrodes in somatosensory cortex are presented elsewhere^46^. Data was collected over 3 testing days between days 661 and 673 after implantation.

### Neural recording and BCI decoder calibration

Neural recording was completed using a Neuroport Neural Signal Processor (Blackrock Microsystems, Inc., Salt Lake City, Utah). Signals were band pass filtered from 250–7500 Hz and digitized. Spikes (i.e. action potentials) were identified using a negative threshold of 4.5 root mean squared (RMS) voltage. All threshold crossings from a single electrode were considered to be a ‘neural unit’ and this may have included both single and multi-neuron recordings. Spike counts on each recording channel were binned in 30 ms bins (33 Hz update rate) for Subject 1 and 20 ms bins (50 Hz update rate) for Subject 2. Binned spike counts were smoothed with an exponential filter with a 450 ms window size for Subject 1 and 440 ms window size for Subject 2. Finally, the firing rates were square root transformed before being used for decoding or data analysis.

As with our previous studies^4,8^, the BCI decoder was trained by having the subject attempt to move a real or virtual robotic arm to a series of position, orientation, and grasp targets. The subjects typically controlled 5 degrees of freedom (DoF) during the experiments described in this protocol. Decoders were trained in virtual reality (VR)^8^ using a two-step calibration method as reported previously^4,8^. The subject attempted to move the VR arm to a presented target location, orient the wrist in accordance with a computerized verbal instruction, and then grasp or release the hand according to a verbal target.

During the first step of calibration, called observation, the subject observed and attempted to execute the arm and hand movements in VR while the computer controlled the actual movements. After completing 27 trials (approximately 6 minutes) of the observation step, an OLE decoder was derived using an encoding model relating the neural firing rates to the arm kinematics. The encoding model is:

1 $$f={b}_{0}+{b}_{x}{v}_{x}+{b}_{y}{v}_{y}+{b}_{z}{v}_{z}+{b}_{r}{v}_{r}+{b}_{g}{v}_{g}$$

where *f* is the square-root transformed firing rate of a unit, *v* is a kinematic velocity, and *b* is a regression coefficient for a given velocity dimension. The dimensions shown here are *x*, *y*, and *z* translation, wrist roll (*r*), and grasp aperture (*g*). The *b* coefficients were calculated using linear regression with ridge regression. Decoder weights were then calculated using indirect optimal linear estimation^4^.

Once this decoder was trained, the subject was given control of the virtual arm for the second step of calibration. During this step, the subject performed the task with the BCI but shared mode control was applied in order to project the decoded command signals onto an idealized path as demonstrated by Velliste *et al*.^19^ to prevent the need for error correction. After completing 27 trials (approximately 6 minutes) of the shared mode control step of training a new decoder was computed. This new decoder was then used, without any form of computer assistance, to complete the tasks described here. For all of the tasks, the subjects controlled 5 dimensions of the modular prosthetic limb (MPL, Johns Hopkins University Applied Physics Laboratory)^47^. This included 3-dimensional endpoint velocity that allowed them to position and move the hand anywhere in the workspace of the robot as well as 1-dimensional rotation of the wrist and closing or opening velocity of hand aperture at all times. The thumb and fingers were linked together into a single dimension of grasp.

### Investigation of cortical activity related to object presence

**Figure 4:**
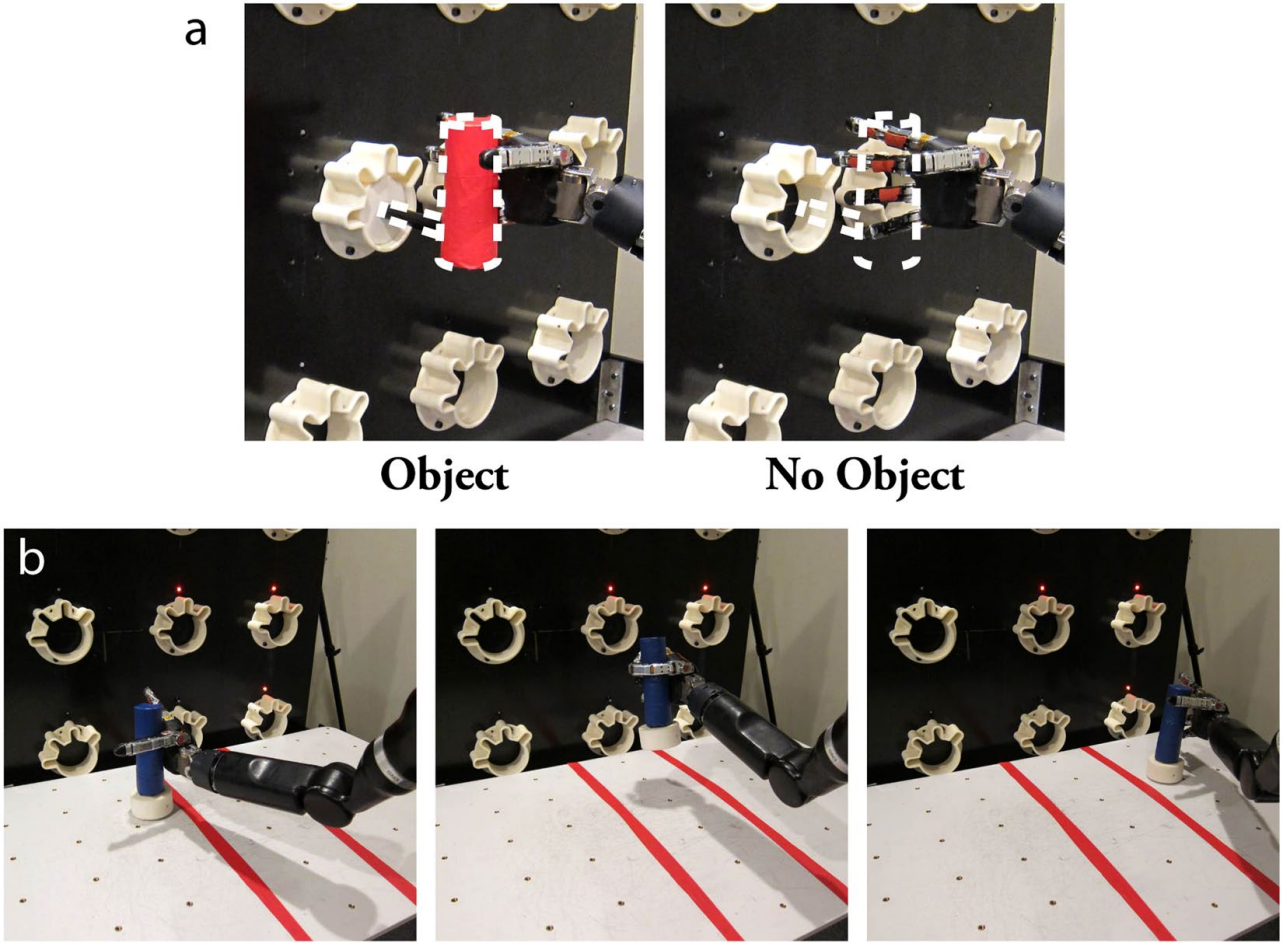
Object interaction and transport tasks. (**a**) The object interaction task required the subjects to move to a position target on the left side of the workspace before closing the hand. This task was repeated with and without and object in the workspace. (**b**) The object transport task required the subjects to grasp the cylindrical object on the left side of the table, carry it over the taped off region, and place it on the right side of the table as many times as possible within two minutes.

The ‘Object interaction task’ was used to determine how the presence of an object impacted neural activity during reaching and grasping. Each subject completed two conditions: (1) Reaching to and grasping a cylindrical object (5 cm diameter, 14 cm height) and (2) Reaching to the same position in space and closing the hand in the absence of a physical object in the workspace (Fig. 4a). The robotic arm started at the right side of the workspace for each trial, which was divided into two phases, reach and grasp. An audio cue indicated the start of the trial and the subjects used the BCI to move the robotic arm to within 5 cm of the target that was 35 cm to the left of the starting position. The same position target was used to judge the success of the reach phase regardless of whether an object was in the workspace. The subject had 20 seconds to reach to the target. Once the hand was within the target area, the grasp phase began as a chime prompted the subject to close the fingers to a grasped position while remaining within 5 cm of the arm position target. The grasp had to be held for 2 continuous seconds within 5 seconds of the chime for the trial to be successful. Data was collected in blocks of 5 trials, where the presence or absence of the object was randomized between blocks. Subject 1 also performed a version of this task where the hand had to be held in an open posture (i.e. finger extended) instead of grasping the fingers closed. Trial completion success rates were compared as a functional measure of the difficulty of object interaction.

For offline analysis, the binned, filtered and square root transformed spike counts (see “Neural recording”) were converted to z-scores on a per channel basis using the means and standard deviations of firing rates observed during calibration. Data was z-scored in order to compare changes in activity between units with different baseline firing rates. Neural data was aligned to the end of the reach phase, which occurred when the position target at the left side of the workspace was achieved. We used the last 1.5 seconds of the reach phase, just before the hand reached the target position, and the first 1.5 seconds of the grasp phase, which occurred after the hand reached target position but before the 2 second hold time could elapse. We tested for differences in the firing rates of the recorded population in the two different test conditions.

### Implementation and evaluation of online firing rate scaling

Since we had previously observed differences in BCI performance when an object was in the workspace^8^, we implemented a method for accounting for observed changes in firing rate. These firing rate changes, as described in more detail in the Results, were broadly distributed across the neural population so an online population-level scaling factor was implemented. The scaling factor was calculated by dividing the average population firing rate during the previous 300 ms by the average population firing rate during decoder calibration as shown below:

2 $$Scaling\,Factor=\,\frac{Average\,Population\,Firing\,Rate\,Over\,Previous\,300\,ms}{Average\,Population\,Firing\,Rate\,During\,Decoder\,Calibration}$$

The firing rate of each channel was then divided by this scaling factor before being decoded to generate kinematic commands.

The subject repeated the object interaction task with online scaling to evaluate whether BCI performance improved with objects in the workspace. The decoder remained constant, but scaling was turned on and off pseudorandomly between blocks of 5 trials. We also evaluated the subjects’ ability to perform a BCI-controlled object transport task with and without the online scaling feature. The goal of the task was to pick up a cylindrical object (15.5 cm tall, 4.5 cm diameter, with a weighted base) on one side of a table, transport it across a 20 cm wide taped off zone on the table, and place it on the other side (Fig. 4b). An experimenter immediately picked up the object and placed it back in the starting position. The subject had to transport the object as many times as possible in 2 minutes. Performance was quantified as the average number of transfers per minute. This task was modeled after the Box and Block^48^ task, but uses just a single, larger object. For both tasks, the subjects were not told whether scaling was on or off for a given block of trials. Subject 1 completed the task 17 times over 6 days of testing. Subject 2 completed the task 20 times over 3 days of testing.

Deidentified datasets generated during the current study are available from the corresponding author on reasonable request.

## Electronic supplementary material

Supplementary information

Supplementary Video 1

Supplementary Video 2

Supplementary Video 3

Supplementary Video 4

Supplementary Video 5
