# Supplementary material for: Motor cortical activity changes during neuroprosthetic-controlled object interaction
Source: Sci Rep. 2017 Dec 5;7:16947. doi: 10.1038/s41598-017-17222-3 (PMC5717217; doi:10.1038/s41598-017-17222-3)
Supplement: Supplementary file 1 — Supplementary information [file 41598_2017_17222_MOESM1_ESM.docx]

**Motor cortical activity changes during neuroprosthetic-controlled object interaction**

John E. Downey, Lucas Brane, Robert A. Gaunt, Elizabeth C. Tyler-Kabara, Michael L. Boninger, Jennifer L. Collinger

Supplementary Video 1. Undesired movements when the hand is near an object. On three different days, Subject 1 had different difficulties grasping an object. In the first example, she was unable to close the hand around the object, but could close the hand when she moved away from the object. In the second example, she struggled to keep the hand open until she reached the object, but she was able to open it multiple times after moving away from the object. In the final example, she was unable to keep the hand still near the target for long enough to grasp it, instead knocking it over after an otherwise normal reaching movement.

Supplementary Video 2. Difference in object interaction task performance when reaching to an object. During reaches to the object (trials on the left) are Subject 2 cannot stabilize the hand in the target region as well as in reaches to no object (trials on the right).

Supplementary Video 3. Difference in object interaction task performance with scaling. When reaching to an object with scaling (trials on the right) Subject 2 can stabilize the hand in the target region better than without scaling (trials on the left). This results in better object grasping. Both sets of trials were collected on the same day using the same decoder.

Supplementary Video 4. Difference in object transfer performance with scaling for Subject 1. Subject 1 transferred the object more quickly with scaling (trials on the right) than without scaling (trials on the left). These were consecutive trials with the same decoder, only scaling was changed between trials.

Supplementary Video 5. Difference in object transfer performance with scaling for Subject 2. Subject 2 transferred the object more quickly with scaling (trials on the right) than without scaling (trials on the left). These were consecutive trials with the same decoder, only scaling was changed between trials.
